# A Prospective Cohort Study of Metformin as an Adjuvant Therapy for Infertile Women With Endometrial Complex Hyperplasia/Complex Atypical Hyperplasia and Their Subsequent Assisted Reproductive Technology Outcomes

**DOI:** 10.3389/fendo.2022.849794

**Published:** 2022-06-30

**Authors:** Wei-ya Kong, Zheng-ai Liu, Na Zhang, Xue Wu, Xing-bo Zhao, Lei Yan

**Affiliations:** ^1^ School of Medicine, Cheeloo College of Medicine, Shandong University, Jinan, China; ^2^ Department of Reproduction, Maternal and Child Health Hospital of Zoucheng, Zoucheng, China; ^3^ Shandong Provincial Hospital Affiliated to Shandong First Medical University, Jinan, China

**Keywords:** complex hyperplasia, complex atypical hyperplasia, progesterone, metformin, assisted reproductive technology

## Abstract

**Objective:**

To investigate the adjuvant efficacy of metformin treatment to achieve pathological complete response (CR) in patients with endometrial complex hyperplasia (CH) and complex atypical hyperplasia (CAH), and secondarily, to evaluate their pregnancy outcomes after following assisted reproductive technology (ART).

**Study Design:**

This prospective cohort study analyzed 219 patients diagnosed with infertility and CH/CAH from January 2016 to December 2020. Among these patients, 138 were assigned to the control group (progesterone alone) and 81 were assigned to the study group (progesterone+metformin). After 8/12 weeks of therapy, the treatment responses were assessed by histological examination of curettage specimens obtained by hysteroscopy. Once the pathological results indicated CR, the patients were able to receive ART. The ART treatment and follow-up data of these patients were collected and analyzed.

**Results:**

116 patients in the control group achieved CR, compared with 76 patients in the study group. The CR rate in the control group was significantly lower than that in the study group (P=0.034). We then divided the patients into subgroups to compare the treatment responses. In the subgroup analyses, patients with body mass index (BMI) ≥25 kg/m^2^ and patients with polycystic ovarian syndrome (PCOS) had higher CR rates in the metformin group compared with the control group (P=0.015, P=0.028 respectively). Subsequently, 68 patients in the control group and 47 patients in the study group received an ART cycle. We examined the pregnancy indications and found no significant differences in the clinical pregnancy rate and live birth rate between the two groups (P>0.05).

**Conclusion:**

Regression of CH/CAH may be improved by progesterone+metformin compared with progesterone alone. The effect was particularly pronounced in patients with BMI ≥25 kg/m^2^ and patients with PCOS. Metformin had no obvious effect on subsequent ART outcomes. The trial is registered on the publicly accessible website:

**Clinical Trial Registration:**

http://www.chictr.org.cn/showproj.aspx?proj=15372, identifier ChiCTR-ONR-16009078.

## Introduction

Endometrial complex hyperplasia (CH) and complex atypical hyperplasia (CAH) are proliferative lesions that can be precursors of endometrial cancer (EC) and mainly manifest as abnormal uterine bleeding, endometrial thickening, and infertility. Histologically, they are characterized by marked glandular complexity and crowding producing a back-to-back appearance (1). Performance of a hysterectomy is recommended for CAH patients (2). Whereas, the traditional treatment of oral progesterone therapy plays a dominant role in fertility preservation, although oral progesterone does not necessarily work in all patients and is associated with a high recurrence rate (3–7).^1^Thus other adjuvant therapy for women diagnosed with infertility and CH/CAH is required.

Metformin, which acts as an insulin sensitizer and is most commonly used in type 2 diabetes mellitus, was confirmed to be an effective treatment for various cancers in human studies (8–12). Metformin has also been used to reverse endometrial hyperplasia and reduce the incidence of EC (12–15), and has no adverse effects on fertility compared with existing treatments (typically progestogens, eg, oral or intrauterine) (15). Furthermore, metformin was shown to inhibit disease relapse after progesterone treatment (16). Previous studies showed that patients in different body mass index (BMI) ranges responded differently to medication (17, 18). Moreover, a study indicated that metformin may be beneficial for PCOS and obesity (19). And a review demonstrated the metformin therapy is more effective in the severely overweight and obese patients in women with PCOS (20). 

Clinical trials of hormonal therapies and definitive standard treatments remain to be established for the management of CH/CAH. Introduction of infertility treatment including assisted reproductive technology (ART) soon after achievement of complete response (CR) would be beneficial for patients (6, 21). In our previous study, we evaluated fertility and oncologic outcomes in women with CH/CAH who received fertility-sparing therapy and *in vitro* fertilization (IVF) and suggested that oral progestin was a useful temporizing treatment and IVF was effective (22).

Nevertheless, at present, there is insufficient evidence to support or refute the use of metformin for treatment of CH/CAH (23). A updated review on metformin versus placebo/no treatment before or during IVF/intracytoplasmic sperm injection(ICSI) treatment in women with polycystic ovary syndrome(PCOS) found no conclusive evidence that metformin improved the live birth rate (24). Whereas, metformin treatment was demonstrated to improve the efficiency of IVF and was considered a supplementary drug in ART in another review (20).

Based on the above research findings, we proposed the hypothesis: metformin combined with progesterone may had a synergistic role in the treatment of CH/CAH, thus improved regression of CH/CAH compared with progesterone alone. Patients with obesity and PCOS were more likely to benefit from metformin. And metformin had uncertain effect on subsequent ART outcomes. In the present study, we evaluated the adjuvant therapeutic effect of metformin on the treatment of CH/CAH and observed its influence on subsequent ART pregnancy outcomes.

## Methods

### Study Design

This prospective cohort study was conducted at the Reproductive Hospital Affiliated to Shandong University. The code for the study in the Chinese clinical trial registry is CHICtr-ONR-16009078. Initially we intended to conduct a randomized controlled trial. With the study going on, there was a period of time when there was no drug for medroxyprogesterone acetate(MPA) in many hospitals including ours, and megestrol acetate(MA) was used instead. Moreover, the dosage and duration of drugs were slightly inconsistent between the two groups. It could not be carried out in strict accordance with a randomized control trial, so it was changed to a prospective cohort study. Institutional review board approval for the study was obtained from the Ethics Committee of the hospital (No. 2015-20). All patients signed informed consent before participating in the research and grouping. The enrolled patients were followed and an ART cycle was subsequently performed after the patients achieved CR. Data on age, BMI, treatment response, and reproductive outcomes were collected from an electronic medical record system and by telephone follow-up.

### Study Population

Patients who were diagnosed with infertility first and then pathologically confirmed CH/CAH after hysteroscopic surgeries between January 2016 and December 2020, had a desire for preservation of fertility, and adhered to the treatment and follow-up were enrolled. Infertility was defined as the absence of pregnancy with unprotected intercourse for one year, and often divided into primary and secondary infertility types. Infertility diagnoses included female ovulatory dysfunction, tubal blockage, male asthenospermia, teratospermia, and unexplained infertility, these patients had one or more diagnoses above-mentioned. The exclusion criteria were: (1) recent administration of hormone therapy within 12 weeks; (2) presence of metformin and oral progesterone contraindications; and (3) presence of chronic diseases affecting the heart, liver, kidney, and other important organs.

### Study Procedures

After power analysis of sample sizes, the total 219 analyzed patients were assigned into two groups according to the willingness of patients and personal preference of different doctors. Single agent therapy with oral progestin alone was defined as control group(Prog group). Based on the severity of pathology, progesterone drug specification, and doctor preference, one treatment method was selected from these three to conduct: 160 mg of MA daily, 250 mg MPA daily, and 500 mg of MPA daily. Combination therapy with progesterone and metformin was defined as study group(Prog+Met group), patients who received the above doses of oral progesterone plus 500 mg of metformin thrice daily. Patients received treatment for 8 or 12 weeks in accordance with their pathology. After taking the medicine, their treatment responses were assessed by histological examination of curettage specimens obtained by hysteroscopy. If the disease was completely cured, subsequent ART was suggested; if the treatment failed or the disease progressed, the conservative treatment could be terminated after consultation with the patient and her family.

The patients were recommend to receive IVF/ICSI due to female oviduct factors, male factors,or both. And preimplantation genetic test(PGT) were selected for patients combined with genetic factors, such as chromosomal structural abnormalities, and monogenic diseases. Most of those who were cured underwent IVF/ICSI/PGT using standard controlled ovarian stimulation (COS) protocols. During ovarian stimulation, monitoring of ovarian response, adjustment of Gonadotropin (Gn) dose, oocyte retrieval, and fertilization were performed as previously described (25). Embryos were scored according to the morphologic criteria of Puissant et al. (26). High-quality embryos were defined as Day 3 embryos with 6-10 cells, even cleavage, and <10% cytoplasmic fragments. On day 3, one or two high-quality embryos were picked out for fresh transfer. Day-5 embryo transfers were performed in case of poor embryo quality or at the request of the patient. Subsequently, luteal support regimens were implemented in accordance with our hospital standards. All pregnancies were followed up at fixed times. Meanwhile, some patients had frozen embryos before the onset of CH/CAH or refused the ART treatment, and their reproductive outcomes were thus outside the scope of the evaluation.

### Study Outcomes

The primary outcome of the study was regression of CH/CAH, including the complete and partial response rates. CR was defined as reversion of CH/CAH to proliferative or secretory endometrium. Partial response (PR) was defined as regression of CAH to simple or complex hyperplasia without atypia, or regression of CH to simple hyperplasia. No response (NR) was defined as unchanged lesion from the initial diagnosis, and progressive disease (PD) was defined as any appearance of endometrial malignancy in all patients or any appearance of CAH in CH patients. The CR/PR/NR/PD rate was defined as the number of patients with CR/PR/NR/PD divided by the total number of patients in the group. It should be noted that only responses of the first review were collected, and data collected of subsequent treatment were not evaluated in the study. Moreover, concerned about the previous conclusion that metformin may be more efficacious for patients with BMI ≥25 kg/m² (17), we divided patients into two subgroups according to BMI: Overweight BMI, ≥25 kg/m^2^ and normal weight BMI, <25 kg/m^2.^ Patients were also divided into other subgroups according to age, and PCOS to compare the CR rate.

Secondary outcome measures included the adverse effects during treatment(including dizziness, drowsiness, and gastrointestinal reactions), and ART treatment outcomes such as clinical pregnancy rate (the number of women was detected gestational sac on ultrasonography divided by the number of embryos transfer cycles), abortion rate (spontaneous loss of clinical pregnancy before 28 weeks of gestation divided by the number of clinical pregnancies)and live birth rate(the number of cycles delivered after 28 weeks of gestation and got live birth divided by the number of embryo transfer cycles).

### Sample Size Calculation

Based on the preliminary data from our IVF clinic and the literature reported data, the complete and partial remission rates of MPA treatment was about 70%. The groups were designed to be allocated on a 1:1 ratio. We aimed to test a difference of 15% between the two groups (i.e. 90% in the Prog+Met group and 75% in the Prog group) with a statistical power of 80% (α=0.05, β=0.2). The minimal sample size calculated was 69 for each group with a dropout rate of 10%.We enrolled more patients than we had planned because the variation of the study design.

### Statistical Analysis

SPSS 24.0 software (IBM Corp., Armonk, NY) was used for statistical analysis of relevant data. Measurement data with a normal distribution were expressed as mean ± standard deviation. An independent-sample t-test was used for comparisons between the two groups. Measurement data that did not conform to a normal distribution were represented by median (25th percentile, 75th percentile). The Mann-Whitney rank-sum test was used for comparisons between the two groups. Counting data were expressed as percentage, and intergroup comparisons were performed using the chi-square test or Fisher’s exact test. Multivariate analysis was performed using the multivariate ordinal logistic regression model to adjust for potential confounders. The treatment outcome(CR-PR-NR-PD) was used as the dependent variable. A total of 5 clinically significant factors were used as the independent variables. In which the categorical variables included progestin types and dosages(MA 160mg/d, MPA 250mg/d or MPA 500mg/d), treatment durations(8 weeks or 12 weeks), whether PCOS is combined and whether metformin is added; continues variable was only BMI in kg/m^2^. Odds ratio with 95% confidence interval was calculated for each of independent variables. Values of P<0.05 were considered to indicate statistical significance.

## Results

**Figure 1:**
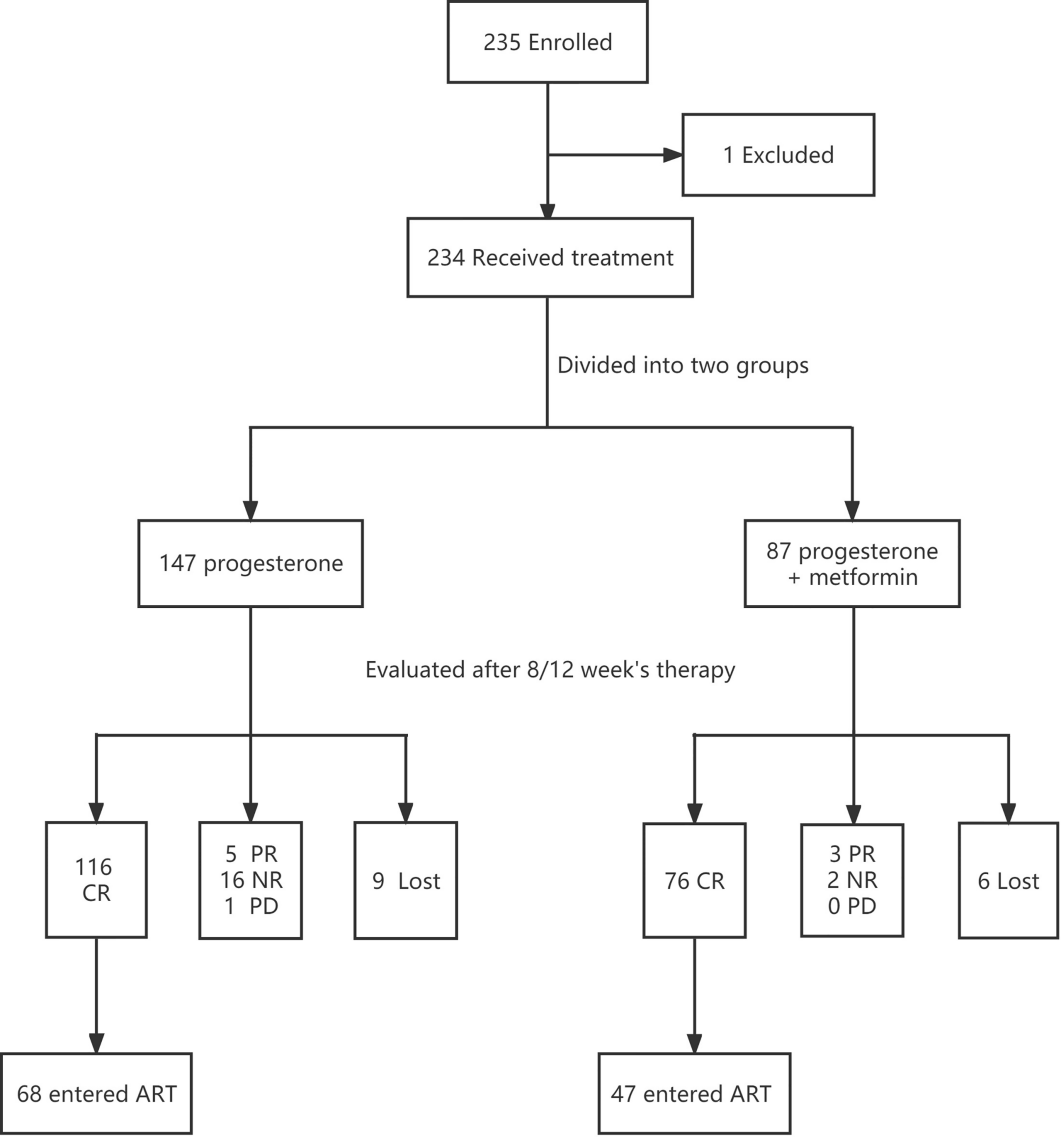
Study flow chart.

A total of 235 patients were initially enrolled. One patient was discontinued and switched to other treatment due to a drug allergy. There were 9 patients in control group and 6 patients in study group did not finish follow-up in our hospital due to distance and COVID-19 pandemic. Finally 219 patients adhering to treatment were analyzed in our study including 138 assigned to the Prog group and 81 assigned to the Prog+Met group. After 8/12 weeks of treatment, 116 patients achieved CR in the control group and among whom 68 agreed to enter an ART cycle. Meanwhile, 76 patients in the study group achieved CR, of whom 47 entered an ART cycle. The patients who received ART were scheduled to receive IVF/ICSI/PGT treatment according to their indications(for example, ICSI for male factors, PGT for chromosomal factors). We followed up their treatment and pregnancy outcomes and compared the two groups. **Figure 1** shows a flow chart of the whole study.

The basic characteristics of the two groups are detailed in **Table 1**. There was no significant difference in mean age between the two groups. BMI and fasting blood glucose after an overnight fast also did not differ significantly between the two groups. The proportions of patients with PCOS, primary infertility, history of CH/CAH(having experienced the same disease,namely, previously diagnosed CH/CAH before the present study), and pathologically confirmed CAH were comparable between the two groups. Additionally, progestin treatment regimens and durations were equally distributed between the two treatment cohorts.

**Table 1:** Baseline characteristics of the patient.

	Prog	Prog+Met	P-value
	N = 138 patients	N = 81 patients	
Age (years), mean ± SD	33.05 ± 5.14	32.00 ± 4.58	P=0.130
BMI (kg/m2), mean ± SD	26.33 ± 4.30	27.01 ± 4.43	P=0.273
FBG (mmol/L), mean ± SD	5.50 ± 0.69	5.59 ± 0.82	P=0.368
PCOS, n(%)	43 (31.2%)	30 (37.0%)	P=0.373
Primary infertility, n(%)	96 (69.6%)	57 (70.4%)	P=0.900
History of CH/CAH, n(%)	12 (8.7%)	9 (11.1%)	P=0.558
CAH, n(%)	49 (35.5%)	29 (35.8%)	P=0.965
Types and dosages of progesterone			P=0.181
MA 160mg/d, n(%)	23 (16.7%)	22 (27.2%)	
MPA 250mg/d, n(%)	50 (36.2%)	24 (29.6%)	
MPA 500mg/d, n(%)	65 (47.1%)	35 (43.2%)	
Treatment durations, n(%)			P=0.814
8 weeks	84 (60.9%)	48 (59.3%)	
12 weeks	54 (39.1%)	33 (40.7%)	

Prog, progesterone; Met, metformin; SD, standard deviation; BMI, body mass index; FBG, fasting blood glucose; PCOS, polycystic ovarian syndrome; CH, complex hyperplasia; CAH, complex atypical hyperplasia; MA, megestrol acetate; MPA, medroxyprogesterone acetate.

The main outcome indicators of the study are shown in **Table 2**. Through data collection and statistical analysis, we found that the rates of effective treatment and CR were lower in the Prog group compared with the Prog+Met group (87.7% vs. 97.5%, P=0.024; 84.1% vs. 93.8%, P=0.034). Meanwhile, the NR rate was higher in the Prog group than in the Prog+Met group(11.6% vs. 2.5%, P=0.034). The rate of PD were similar between the two groups (P=1.000). We also recorded the adverse reactions in the two groups after taking the medications. The main symptoms included dizziness, nausea and vomiting, breast-distending pain, while no irreversible toxicities were observed. The two groups did not differ significantly in the incidence ratios of these adverse effects (0.7% vs. 4.9%, P=0.122).

**Table 2:** Comparison of treatment responses between the Prog group and the Prog+Met group.

	Prog	Prog+Met	P-value
	N = 138 patients	N = 81 patients	
CR+PR, rate, %(n)	87.7% (121/138)	97.5% (79/81)	P=0.024
CR,rate, %(n)	84.1% (116/138)	93.8% (76/81)	P=0.034
NR,rate, %(n)	11.6% (16/138)	2.5% (2/81)	P=0.034
PD, rate, %(n)	0.7% (1/138)	0% (0/81)	P=1.000
Incidence of adverse effects, %(n)	0.7% (1/138))	4.9% (4/81)	P=0.122

Prog, progesterone; Met, metformin; CR, complete response; PR, partial response; NR, no response; PD, progressive disease.

Whereafter, multivariate analysis was performed using the multivariate ordinal logistic regression model to adjust for potential confounders. Several factors of clinical significance were taken into account, including progestin types and dosages, treatment durations, BMI, PCOS and metformin. These data in **Table 3** indicated that metformin was an independent influence factor for the treatment outcome (P=0.017).

**Table 3:** Multivariate analysis of potential confounders associated with the treatment response.

Factors	OR	95%CI	P-value
Types and dosages of progesterone			
MA 160mg/d	2.565	0.962-6.844	P=0.060
MPA 250mg/d	0.781	0.247-2.474	P=0.674
MPA 500mg/d	1		
Treatment durations (8 weeks or 12 weeks)	0.524	0.213-1.292	P=0.161
BMI (kg/m^2^)	1.027	0.929-1.134	P=0.606
PCOS	1.559	0.652-3.729	P=0.318
Met	0.274	0.095-0.792	P=0.017

OR, odds ration; CI, confidence interval; MA, megestrol acetate; MPA, medroxyprogesterone acetate; BMI, body mass index; PCOS, polycystic ovarian syndrome; Met, metformin.

Next, we divided the patients into subgroups according to age, BMI, and PCOS, and examined whether there were any differences in the treatment effects between the study group and the control group among the different subgroups. As shown in **Table 4**, no significant differences in response according to different ages were found between the two groups. Notably, the results showed that patients with BMI ≥25 kg/m^2^ and patients with PCOS had significantly higher CR rates in the Prog+Met group compared with the Prog group (P=0.015, P=0.028).

**Table 4:** Comparison of treatment responses between the two groups in subgroup analyses.

	Prog	Prog+Met	P-value
	N = 138 patients	N = 81 patients	
Age and CR rate			
≥35 years old, %(n)	83.0% (39/47)	96.0% (24/25)	P=0.224
<35 years old, %(n)	84.6% (77/91)	92.9% (52/56)	P=0.222
BMI and CR rate			
≥25, %(n)	77.2% (61/79)	94.4% (51/54)	P=0.015
<25, %(n)	93.2% (55/59)	92.6% (25/27)	P=1.000
PCOS and CR rate			
PCOS, %(n)	74.4% (32/43)	96.7% (29/30)	P=0.028
NoPCOS, %(n)	88.4% (84/95)	92.2% (47/51)	P=0.672

Pro, progesterone; Met, metformin; CR, complete response; BMI, body mass index; PCOS, polycystic ovarian syndrome.

Patients who achieved CR were subsequently scheduled to receive ART, according to the individual willingness of the patients. As a result, 68 patients in the control group received an ART cycle (56 IVF, 10 ICSI, 2 PGT) as did 47 patients in the study group (41 IVF, 4 ICSI, 2 PGT). **Table 5** shows the COS characteristics in these patients. The total Gn days, Gn dosages, endometrial thicknesses on the trigger day, and ovarian responses to the drug were comparable between the two groups (P>0.05). The two groups did not differ significantly in the numbers of high-quality embryos (P=0.477).

**Table 5:** Characteristics of IVF-ICSI-PGT stimulation cycles and embryological results.

	Prog	Prog+Met	P-value
	N = 138 patients	N = 81 patients	
Number of IVF/ICSI/PGT cycles, n	68	47	
Total Gn time (Day), mean ± SD	9.94 ± 4.00	11.13 ± 3.05	P=0.089
Total Gn dose (IU), mean ± SD	2214.15 ± 1203.50	2380.56 ± 1180.71	P=0.464
EM thickness on trigger-day (cm), mean ± SD	1.01 ± 0.25	0.95 ± 0.22	P=0.144
>14mm Oocytes on trigger-day,mean ± SD	8.76 ± 5.90	8.64 ± 5.92	P=0.910
Oocytes obtained, median (IQR)	6.00 (3.00,13.00)	6.00 (2.00,12.00)	P=0.636
High-quality embryos, median (IQR)	2.00 (0.00,5.00)	2.00 (1.00,4.00)	P=0.477

IVF, in vitro fertilization; ICSI, intracytoplasmic sperm injection; PGT, preimplantation genetic test; Prog, progesterone; Met, metformin; Gn, gonadotrophin; SD, standard deviation; EM, endometrial.

Finally, we compared the reproductive results for fresh embryo transfer cycles between the Prog group and the Prog+Met group. Because the population undergoing fresh embryo transfer was a small subset of the initial population, the characteristics of these patients were compared separately. The baseline factors and the outcomes are presented in **Table 6**. It was feasible to transfer fresh embryos in 38 patients in the Prog group and 27 patients in the Prog+Met group, other patients did not conduct a fresh embryo transfer because of ovarian hyperstimulation syndrome, thin endometrium (thickness<0.75cm) in fresh cycle or PGT. There were no significant differences in mean age and BMI between the two groups. The proportions of patients with PCOS, primary infertility were comparable between the two groups. There were no significant differences in the rates of cleavage day-3 embryo transfer cycles and two-embryo embryo transfer cycles between the two groups (P=0.753, P=0.362). Compared with the control group, the rates of embryo implantation, clinical pregnancy, and abortion in the study group did not differ significantly (P>0.05). In addition, no significant differences were found in the live birth rate, term delivery rate, and neonatal birth weight between the two groups (P>0.05). However, the final sample size was small for the analysis of ART outcomes. It was underpowered to interpret the determination of no difference between study and control groups.

**Table 6:** Baseline factors and reproductive outcomes calculated per fresh ET cycle.

	Prog	Prog+Met	P-value
	N = 138 patients	N = 81 patients	
Number of ET cycles,n	38	27	
Age (years), mean ± SD	32.39 ± 6.37	31.70 ± 4.66	P=0.633
BMI (kg/m2), mean ± SD	26.04 ± 4.26	27.34 ± 3.93	P=0.216
PCOS, n(%)	9 (23.6%)	10(37.0%)	P=0.243
Primary infertility, n(%)	28 (73.7%)	18(66.7%)	P=0.540
EM thickness on trigger-day(cm), mean ± SD	0.98 ± 0.20	0.93 ± 0.21	P=0.377
Cleavage day-3 ET cycles, %(n)	78.9% (30/38)	85.2% (23/27)	P=0.753
2 embryos ET cycles, %(n)	63.2% (24/38)	51.9% (14/27)	P=0.362
Implantation rate, %(n)	27.4% (17/62)	29.3% (12/41)	P=0.838
Biochemical pregnancy rate, %(n)	47.4% (18/38)	48.1% (13/27)	P=0.951
Clinical pregnancy rate,%(n)	36.8% (14/38)	37.0% (10/27)	P=0.987
Abortion rate, %(n)	35.7% (5/14)	40.0% (4/10)	P=1.000
Live birth rate, %(n)	23.7% (9/38)	22.2% (6/27)	P=0.890
Term delivery rate, %(n)	77.8% (7/9)	83.3% (5/6)	P=1.000
Neonatal birth weight(kg), mean ± SD	3.12 ± 0.55	3.20 ± 0.79	P=0.816

ET, embryo transfer; Prog, progesterone; Met, metformin; SD, standard deviation; BMI, body mass index; PCOS, polycystic ovarian syndrome; EM,endometrial.

## Discussion

The subjects in this study were infertile patients with CH/CAH, for whom the oral progesterone is widely prescribed as the preferred hormone treatment, because it can maximize protection of the endometrium. However, oral progesterone has many limitations and the need to find adjuvant drugs has led to the use of metformin. In the present study, adjunctive metformin therapy outperformed progestin monotherapy in the fertility-sparing treatment of CH/CAH patients, with a significantly higher CR rate in the Prog+Met group compared with the Prog group, especially in the subgroup analyses for patients with BMI ≥25 kg/m^2^ and patients with PCOS. We compared the pregnancy outcomes in the Prog group and the Prog+Met group, and found no significant differences in the biochemical pregnancy rate, clinical pregnancy rate, and live birth rate between the two groups.

Previous studies on oral progesterone treatment for CAH found CR rates of 74% to 88.9% (3, 4, 27, 28). Our results also confirmed the effect of oral progesterone is undisputed. Potential side effects of oral progestin therapy includes headache, mood changes, and an increased risk of thromboembolic events or breast cancer in the long term. The optimal dosage and duration of treatment are unclear (29, 30). A review showed that CR of CH/CAH was achieved in a lower proportion of women treated with oral progestogens compared with women treated with levonorgestrel intrauterine system(LNG-IUS) (31), but LNG-IUS may not accepted by all infertile patients because of its invasive nature and patient desire to attain pregnancy as quickly as possible. A meta-analysis indicated that LNG‐IUS use may be associated with more bleeding/spotting (32). Meanwhile, the rate of disease recurrence after progesterone therapy alone is high (3–6, 27, 28).

The development of CH/CAH is related to potential risk factors such as obesity, diabetes mellitus, and PCOS, similar to the case for EC (33–35). The mean BMI and proportion of PCOS in our patients were clearly higher than those in the normal population. In relation to the risk factors mentioned above, we speculate that chronic hyperinsulinemia secondary to insulin resistance may have a direct mitogenic effect on the endometrium, as a risk factor for hyperplasia and even carcinoma, and may inhibit progestogen therapy (36). A retrospective investigation on 151 atypical hyperplasia(AH) patients draw similar conclusions that insulin resistance and overweight were associated with longer progestin therapeutic duration (37). As an insulin sensitizer, metformin decreases insulin resistance through inhibition of hepatic gluconeogenesis. The insulin-mediating effects of metformin have shown evidence of reducing the incidence of malignancies and improving patient survival (12, 35, 38). In addition, metformin has the potential to increase progestin-driven anti-proliferative mechanisms (39).

Not only has the above mechanism been demonstrated, but some previous clinical trials have corroborated the effectiveness of metformin in inducing endometrial atrophy. One study reported atrophy and therefore reversal of endometrial hyperplasia in 95.5% of women treated with metformin (40). A trial demonstrated that metformin plus MA was associated with a higher early CR rate compared with MA alone in AH patients (41). It is noteworthy that metformin had a significant therapeutic effect on patients with BMI ≥25 kg/m^2^ and patients with PCOS in the present study, this may be explained by metformin effect of decrease insulin resistance was pronounced utilized in these patients. In other studies, metformin was also confirmed to remain significant in non-obese, insulin-sensitive, non-hypertensive, and non-diabetic subgroups of AH patients (41), there may be potential mechanisms which merit further investigation.

Further on, ART was the first option for patients achieved CR, there is evidence that <20% of women treated for AH achieved a live-birth pregnancy, with most requiring ART (42). It could also be due to the patient’s own characteristics, the fertility of present patients will depend on the response to treatment and on underlying patient factors affecting fertility (e.g. the presence of PCOS). Some patients in the study rejected ART for financial and psychological reasons. With regard to pregnancy outcomes, our study confirmed that metformin did not have any effect on the clinical pregnancy and live birth rates during fresh embryo transfer cycles. While one study found that MPA plus metformin was efficacious in post-treatment conception (17). Other studies drew various conclusions, for example, a retrospective cohort study on patients diagnosed AH/EC did not support the use of metformin therapy (42). It is negotiable that whether the limited sample size we obtained analyzing pregnancy outcomes led to some effects undiscovered.

Last but not least, after completion of childbearing, the same risk factors will be present and theoretically continue to predispose the patient to the CH/CAH (30). Therefore, in addition to providing 8/12 weeks of medication, we should give patients lifestyle guidance in the meantime. Thirty-four observational studies that evaluated the outcomes of fertility-sparing treatment indicated a relapse rate of 26% for CAH patients (27). Hysterectomy is advisable after completion of childbirth, given the high recurrence rates after conservative management (6, 28). There is also evidence that up to 50% of women whose endometrial biopsies are classified as AH actually have carcinoma (43).

The strength of the present study is its prospective nature, which is characterized by the relatively controllable medicine dose and review time. However, the formulations of progestin therapy were still varied. It can be noticed that 27.2% of women in the study group vs. only 16.7% of women in the control group were assigned to receive MA, which is more potent than MPA, this limits the interpretation of the study results. And non-randomized design may induce potential bias of grouping. The other limitation of the study is the lack of evaluation of the response rate and recurrence rate beyond 8/12 weeks. In brief, the results of our study can be generally applied in clinical practice. For CH/CAH patients, especially those with obesity and PCOS, progesterone combined with metformin is recommended as a conservative treatment. As for the reproductive outcomes, our data should be reported as an exploratory outcome only because of the small numbers. Future trials with larger sample sizes will hopefully elucidate the best strategy for treatment.

## Conclusions

Metformin had a significant beneficial effect during adjuvant treatment of patients with infertility and CH/CAH, especially for patients with BMI ≥25 kg/m^2^ and patients with PCOS. Metformin had no obvious effect on subsequent ART outcomes in these patients.

## Data Availability Statement

The original contributions presented in the study are included in the article/supplementary material. Further inquiries can be directed to the corresponding authors.

## Ethics Statement

The studies involving human participants were reviewed and approved by the Ethics Committee of the Reproductive Hospital Affiliated to Shandong University. The patients/participants provided their written informed consent to participate in this study.

## Author Contributions

W-yK, NZ, and XW contributed to conception and design of the study. Z-aL and NZ organized the database. X-bZ guided the treatment plan of patients. W-yK performed the statistical analysis. WK wrote the first draft of the manuscript. W-yK, Z-aL, and XW wrote sections of the manuscript. All authors contributed to manuscript revision, read, and approved the submitted version.

## Funding

This study was supported by a grant from the Chinese Medical Association of Clinical Medicine Research Special Funds (No. 16020290645), and the Natural science Foundation of Shandong Province (ZR2018MH027).

## Conflict of Interest

The authors declare that the research was conducted in the absence of any commercial or financial relationships that could be construed as a potential conflict of interest.

## Publisher’s Note

All claims expressed in this article are solely those of the authors and do not necessarily represent those of their affiliated organizations, or those of the publisher, the editors and the reviewers. Any product that may be evaluated in this article, or claim that may be made by its manufacturer, is not guaranteed or endorsed by the publisher.

## Footnotes

1 National Comprehensive Cancer Network (US). Uterine neoplasms [Internet]. Plymouth Meeting, PA: National Comprehensive Cancer Network; c2018 [cited 2018 May 25]. Available from: https://www.nccn. org/professionals/physician_gls/pdf/uterine.pdf

## Abbreviations

CH, complex hyperplasia; CAH, complex atypical hyperplasia; AH, atypical hyperplasia; PR, partical response; PCOS, polycystic ovarian syndrome; ART, assisted reproductive technology; IVF, *in vitro* fertilization; ICSI, intracytoplasmic sperm injection; PGT, preimplantation genetic test; COS, controlled ovarian stimulation; ET, embryo transfer; FET, frozen embryo transfer; Prog, progesterone; Met, metformin; SD, standard deviation; BMI, body mass index; FBG, fasting blood glucose; MA, megestrol acetate; MPA, medroxyprogesterone acetate;NR, no response; PD, progressive disease; Gn, gonadotrophin; EM, endometrial.
